# Alterations in Glomerular Filtration Rates Using Different Algorithms in the Korean Population Visiting Local Clinics and Hospitals

**DOI:** 10.3390/jcm11185339

**Published:** 2022-09-11

**Authors:** Rihwa Choi, Sang Gon Lee, Eun Hee Lee

**Affiliations:** 1 Department of Laboratory Medicine, Green Cross Laboratories, Yongin 16924, Korea; 2 Department of Laboratory Medicine and Genetics, Samsung Medical Center, Sungkyunkwan University School of Medicine, Seoul 06351, Korea; 3 Green Cross Laboratories, Yongin 16924, Korea

**Keywords:** glomerular filtration rate, equation, chronic kidney disease, proteinuria, albuminuria, Korea

## Abstract

We retrospectively reviewed the estimated glomerular filtration rates (eGFR) calculated by three equations: (1) Modification of Diet in Renal Disease Study (MDRD), (2) Chronic Kidney Disease Epidemiology Collaboration (CKD-EPI) using serum creatinine in 2009 (CKD-EPI 2009), and (3) CKD-EPI suggested in 2021 (CKD-EPI 2021), in an adult Korean population visiting local clinics and hospitals for health check-ups between 2019 and 2021 to investigate the effect of changes in the prevalence of chronic kidney diseases using those equations. During the study period, serum creatinine tests were performed on 442,566 subjects (205,236 men and 237,330 women). The overall prevalence rates of decreased eGFR (<60 mL/min/1.73 m^2^) at baseline measurement were as follows: 3.4% using MDRD, 2.8% using CKD-EPI 2009, and 2.1% using CKD-EPI 2021. The prevalence of decreased eGFR increased with age. Among 442,566 tests, specimens having simultaneously measured random urine dipstick tests for proteinuria represented 6.0% of the population, and the albumin-creatinine ratio (ACR) was 0.3%. The prevalence of specimens having both decreased eGFR and proteinuria was significantly different among equations (*p* < 0.05). Among the three equations, MDRD and CKD-EPI 2009 had a similar specimen prevalence of decreased eGFR and proteinuria (≥1+) using a dipstick test or albuminuria (urine ACR > 30 mg/g creatinine), while those based on CKD-EPI 2021 were about half of those based on MDRD and CKD-EPI 2009. Future studies are needed to clarify the clinical impact of the changes in the calculations for eGFR.

## 1. Introduction

Kidney function for the diagnosis and management of chronic kidney disease (CKD) is usually assessed using an estimated glomerular filtration rate (eGFR) [1]. Current criteria for the definition of CKD in adults are: (1) signs of structural or functional kidney damage, including abnormalities in urine test results (albuminuria, sediment abnormalities, electrolytes, and other abnormalities) or imaging or history of kidney transplant, or (2) decreased glomerular filtration rate (GFR, < 60 mL/min/1.73 m^2^) for >3 months [1,2,3,4]. For these criteria, the elevated urine albumin-creatinine ratio (ACR) test is used to assess albuminuria when ACR ≥ 30 mg/g creatinine, and the dipstick urine test using reagent strip urinalysis for total protein with automated reading is commonly used to assess proteinuria in the clinical field [1,2,3,4].

For GFR estimation, several equations have been developed, validated, and used in clinical practice and are estimated from serum concentrations of endogenous filtration markers such as creatinine [5,6,7]. The equations are traceable to isotope dilution mass spectrometry as signed and certified by the National Institute of Standards and Technology reference materials or cystatin C [5]. In 2006, the Modification of Diet in Renal Disease Study (MDRD 2006) equation using standardized serum creatinine level, age, sex, and race was introduced [6]. The Chronic Kidney Disease Epidemiology Collaboration (CKD-EPI) equation using standardized serum creatinine level, age, sex, and race was reported in 2009 (CKD-EPI 2009) [7]. In 2021, a new equation by CKD-EPI using standardized serum creatinine level, age, and sex, without race, was introduced (CKD-EPI 2021) [5]. Since the CKD-EPI 2021 equation has been developed, the effect of the eGFR equations with and without race has been investigated in different ethnic populations [5,8]. However, studies on the effect of the CKD-EPI 2021 equation on the prevalence of CKD in the Korean population have been limited.

Although the current status of CKD patients has been provided through national cohort studies and the factsheet published by the Korea Society of Nephrologists [9], limited data are available for the prevalence of CKD identified through health checkups using large population data. The Korea National Health and Nutrition Examination Survey (KNHANES) investigated the prevalence of CKD defined as (1) both eGFR based on CKD-EPI 2009 ≥ 60 mL/min/1.73 m^2^ and ACR ≥ 30 mg/mg creatinine or (2) eGFR based on CKD-EPI 2009 < 60 mL/min/1.73 m^2^ [10]. Furthermore, the ACR test using standardized creatinine methods was included only for some survey years of KNHANES, from 2011 to 2014 and from 2019 to the current phase (IX). Therefore, recent information on the prevalence of CKD in the general Korean adult population is limited.

Because Green Cross Laboratories performs serum creatinine, dipstick urine protein, and urine ACR tests as requested by local clinics and hospitals throughout Korea, we gathered recent information on the prevalence of patients with possible CKD and the utilization of urine dipstick tests for proteinuria and ACR tests in a large adult Korean population visiting local clinics and hospitals for health checkups. The aim of this study was to investigate the effect of equations for eGFR calculation (MDRD 2006, CKD-EPI 2009, and CKD-EPI 2021) on the specimen prevalence of decreased eGFR with proteinuria or albuminuria as surrogate information on CKD in the adult Korean population.

## 2. Materials and Methods

### 2.1. Subjects

We retrospectively reviewed data obtained through the laboratory information system of Green Cross Laboratories between 1 January 2019 and 31 December 2021 for Korean adults (age > 19 years) who visited local clinics and hospitals and underwent serum creatinine testing for health checkups. Test results were excluded for patients with missing data for age or sex. Repeated measurements in patients were excluded, and only the first measurement of each patient was included for analysis. In order to investigate proteinuria or albuminuria, random urine dipstick tests for protein and urine ACR tests were also reviewed.

### 2.2. Analytical Methods

The serum creatinine level was measured using the Creatinine Jaffe Gen.2 kits (CREJ2; Roche, Mannheim, Germany) based on a kinetic colorimetric assay on c702 chemistry analyzers (Roche, Mannheim, Germany). A random urine dipstick test for proteinuria was performed using Urisys 2400 analyzers (Mannheim, Germany) from 1 January to 30 June 2019 and UC 3500 analyzers (Sysmex, Kobe, Japan) from 1 July 2019 to 31 December 2021. Random urine albumin and creatinine were measured using Tina-quant Albumin Gen.2 kits (ALBT2; Roche, Mannheim, Germany) and CREJ2 kits on a c502 analyzer (Roche, Mannheim, Germany) between 1 January 2019 to 28 June 2020; and Urine/CSF albumin test kits (Beckman Coulter, Galway, Ireland) and CREA kits (Beckman Coulter, Galway, Ireland) on an AU680 analyzer (Beckman Coulter, Tokyo, Japan) from 29 June to 8 November 2020; and on an AU5800 analyzer (Beckman Coulter, Tokyo, Japan) from 9 November 2020 to 31 December 2021. Method comparison tests using clinical specimens between analytical methods were performed. The accuracy of serum creatinine, urine dipstick protein test, urine albumin, and creatinine tests for ACR was assured by participation in the proficiency testing program, including accuracy-based creatinine analysis by the Korean Association of External Quality Assessment Service and by the College of American Pathologists.

### 2.3. Definitions

All data were anonymized before statistical analysis. Patient ages were grouped as 19–29 years, 30–39 years, 40–49 years, 50–59 years, 60–69 years, 70–79 years, and ≥80 years.

Decreased kidney function using eGFR, proteinuria based on dipstick test results, and albuminuria based on ACR tests were determined according to the current clinical guidelines [1,3]. CKD stage based on eGFR is as follows: G1 (normal or high eGFR, ≥90 mL/min/1.73 m^2^), G2 (mildly decreased eGFR, 60–89 mL/min/1.73 m^2^), G3a (mildly to moderately decreased eGFR, 45–59 mL/min/1.73 m^2^), G3b (moderately to severely decreased eGFR, 30–44 mL/min/1.73 m^2^), G4 (severely decreased eGFR 15–29 mL/min/1.73 m^2^), and G5 (kidney failure, <15 mL/min/1.73 m^2^) [1,3]. Albuminuria categories based on the ACR test results are as follows: A1 (normal to mildly increased ACR, <30 mg/g creatinine), A2 (moderately increased ACR, 30–300 mg/g creatinine), and A3 (severely increased ACR, >300 mg/g creatinine) [1,3]. For proteinuria, test results were expressed semi-quantitatively, and reagent strip 1+ protein was defined as proteinuria [1,3].

Equations to calculate eGFR (MDRD 2006, CKD-EPI 2009, and CKD-EPI 2021) are summarized in Supplementary Table S1.

### 2.4. Statistical Analysis

Data are presented as mean and standard deviation for quantitative values and number and percentage for qualitative values. Chi-square tests were used to compare the prevalence of decreased eGFR, proteinuria, and albuminuria status by sex and age group. Linear regression analysis was performed to investigate the correlation between age and eGFR. We investigated the quantitative and qualitative results of the eGFR test results.

We investigated the quantitative and qualitative results of the eGFR test results. Qualitative evaluation for the prevalence of decreased eGFR was investigated using categorical grades of ≤G2, G3a, and ≥G3b by CKD stage guidelines, proteinuria based on dipstick test grade ≥1+, and albuminuria with ACR A1 (<30 mg/g creatinine), A2 (30–300 mg/g creatinine), and A3 (>300 mg/g creatinine) were evaluated by sex and age group. The differences between CKD-EPI 2009 and CKD-EPI 2021 in ml/min/1.73 m2 and as a % were investigated using Bland–Altman plot analysis.

A value of *p* < 0.05 was considered statistically significant with the MedCalc statistical software version 20.110 (MedCalc Software Ltd., Ostend, Belgium).

### 2.5. Ethics

Ethical approval for this study was obtained from the Institutional Review Board (IRB) of Green Cross Laboratories (GCL-2022-1030-02, 16 June 2022). A waiver of informed consent was approved by the IRB because this study was retrospective and involved no more than minimal risk to subjects.

## 3. Results

### 3.1. Baseline Characteristics of Subjects

During the study period, serum creatinine tests were performed in 442,566 subjects (205,236 men and 237,330 women) with a mean age of 50.1 years (SD 15.0). Baseline characteristics of study subjects are summarized in Table 1. Among the age groups, patients aged 40–49 years were the most prevalent (23.9%). Most patients (86.8%, 384,368/442,566) underwent one serum creatinine measurement during the study period.

The overall prevalence rates of decreased eGFR (<60 mL/min/1.73 m^2^) at baseline measurement were as follows: 3.4% using MDRD 2006, 2.8% using CKD-EPI 2009, and 2.1% using CKD-EPI 2021.

### 3.2. eGFR by Age

**Figure 1:**
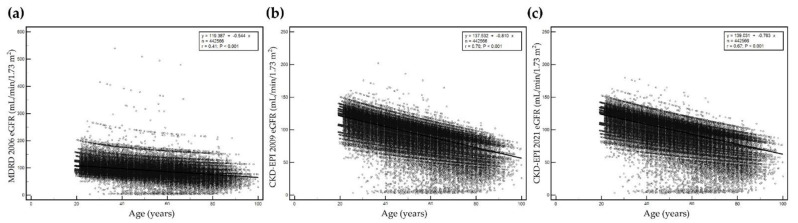
Correlation between age and estimated glomerular filtration rate (eGFR) by age using (**a**) MDRD 2006, (**b**) CKD-EPI 2009, and (**c**) CKD-EPI 2021. Y-axis represents eGFR and x-axis represents age. The maximum scale of the y-axis is 600 mL/min/1.73 m^2^ for MDRD 2006, and 250 mL/min/1.73 m^2^ for both CKD-EPI 2009 and CKD-EPI 2021.

**Figure 2:**
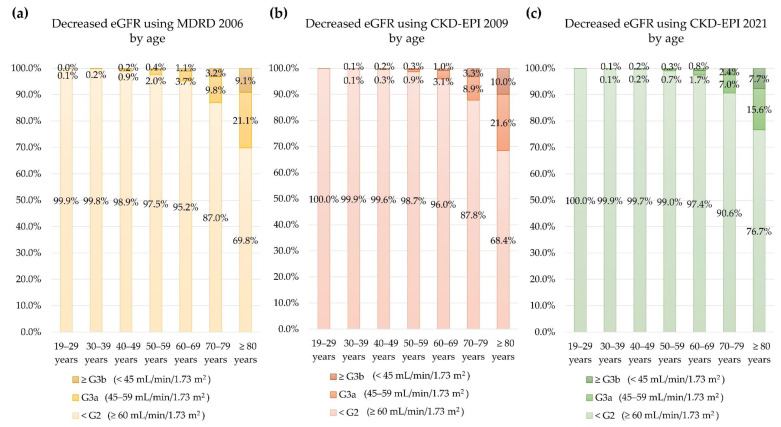
Prevalence of decreased eGFR (<60 mL/min/1.73 m^2^) by age using (**a**) MDRD 2006, (**b**) CKD-EPI 2009, and (**c**) CKD-EPI 2021.

Serum creatinine level, sex, and age at serum creatinine test were used to calculate eGFR. eGFR values were significantly different among equations (*p* < 0.05). Correlations between age and eGFR calculated using each of the three equations are presented in Figure 1. The eGFR values calculated using the three equations decreased with increased age. The correlation coefficient of r was highest for eGFR based on the CKD-EPI 2009 equation (*r* = 0.70, *p* < 0.001) and lowest for eGFR based on the MDRD 2006 equation (*r* = 0.41, *p* < 0.001). The prevalence of decreased eGFR (<60 mL/min/1.73 m^2^) increased with age (Figure 2).

### 3.3. Overall Agreement among the Three Equations

Overall agreement among the three equations is summarized in Table 2. Qualitative categories for decreased eGFR using ≤ G2 (normal to mild decrease, ≥60 mL/min/1.73 m^2^), G3a (mild to moderate decrease, 45–59 mL/min/1.73 m^2^), and ≥G3b (≥moderate decrease, <45 mL/min/1.73 m^2^) based on the three equations are presented. Among the equations, the agreement of eGFR grade between MDRD 2006 and CKD-EPI 2009 and between CKD-EPI 2009 and CKD-EPI 2021 was greater than 99.0%, while the agreement between MDRD 2006 and CKD-EPI 2021 was 98.4%. The levels of eGFR calculated using CKD-EPI 2021 were the highest among the equations (the lowest was eGFR using MDRD 2006). The specimen prevalence of decreased eGFR (≥60 mL/min/1.73 m^2^) using CKD-EPI 2021 (2.0%) was lowest among the three equations, followed by CKD-EPI 2009 (2.8%) and MDRD 2006 (3.4%). The difference between CKD-EPI 2009 and CKD-EPI 2021 in ml/min/1.73 m^2^ and as a % are summarized in Supplementary Figure S1. Mean difference and mean %difference of eGFR level between CKD-EPI 2009 and CKD-EPI 2021 were −3.8 (95% confidence interval −5.8 to −1.9) mL/min/1.73 m^2^ and −4.1% (95% confidence interval −6.8% to −1.3%, respectively). The eGFR from CKD-EPI 2021 was higher than that from CKD-EPI 2009.

### 3.4. Decreased eGFR with Proteinuria or Albuminuria

Among 442,566 tests, specimens having a simultaneously measured random urine dipstick test for proteinuria accounted for 6.0%, and those with an albumin-creatinine ratio (ACR) were 0.3%. Only 319 (0.08%) specimens had a simultaneously measured dipstick test for proteinuria and an ACR test. Among these 319 specimens, 301 (94.4%) were negative for proteinuria (≥1+) or A2 albuminuria (ACR > 30 mg/g creatinine), 15 (4.7%) were positive for both proteinuria and albuminuria, 41 (12.9%) were only positive for albuminuria without proteinuria on the dipstick test, and 3 (0.9%) were only positive for proteinuria without albuminuria.

The prevalence of specimens having decreased eGFR grade and proteinuria or albuminuria is summarized in Table 3. The prevalence of proteinuria (≥1+) using a dipstick test was lower than that of the ≥A2 albuminuria (ACR > 30 mg/g creatinine). Among A1 albuminuria (normal or mild albuminuria, ACR < 30 mg/g creatinine), the prevalence of specimens with decreased eGFR (<60 mL/min/1.73 m^2^) was highest in MDRD 2006 (5.9%) and lowest in CKD-EPI 2021 (3.2%). Among seven specimens having A3 albuminuria (severely increased ACR > 300 mg/g creatinine), the grade of eGFR was the same among the three equations.

**Figure 3:**
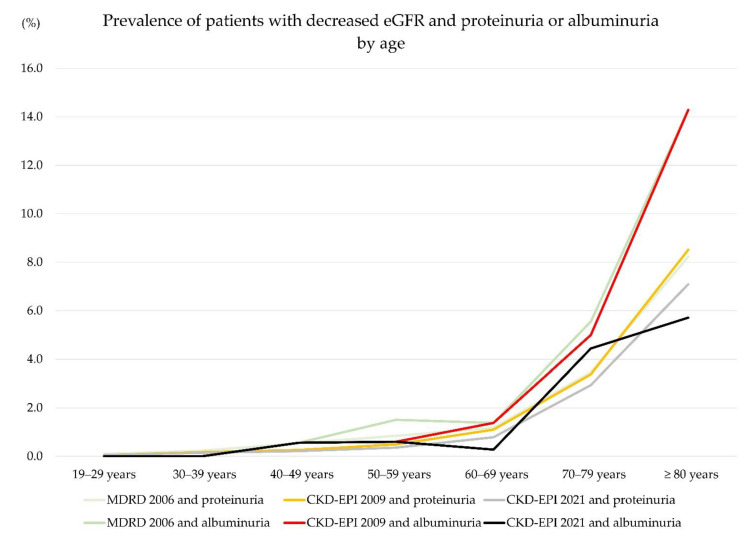
Prevalence of specimens with decreased eGFR (<60 mL/min/1.73 m^2^) and proteinuria (≥1+ in a dipstick urine protein test) or albuminuria (ACR > 30 mg/g creatinine) by age.

The prevalence of specimens having both decreased eGFR (≥G3a, <60 mL/min/1.73 m^2^) and proteinuria or albuminuria was significantly different among equations (*p* < 0.05). The prevalence of specimens having both decreased eGFR (≥G3a, <60 mL/min/1.73 m^2^) and proteinuria or albuminuria is presented in Figure 3. Among the three equations, MDRD 2006 and CKD-EPI 2009 had a similar specimen prevalence of decreased eGFR (≥G3a, <60 mL/min/1.73 m^2^) and proteinuria (≥1+) using a dipstick test or ≥ A2 albuminuria (urine ACR > 30 mg/g creatinine), while that based on CKD-EPI 2021 was about half of that based on MDRD 2006 and CKD-EPI 2009.

**Figure 4:**
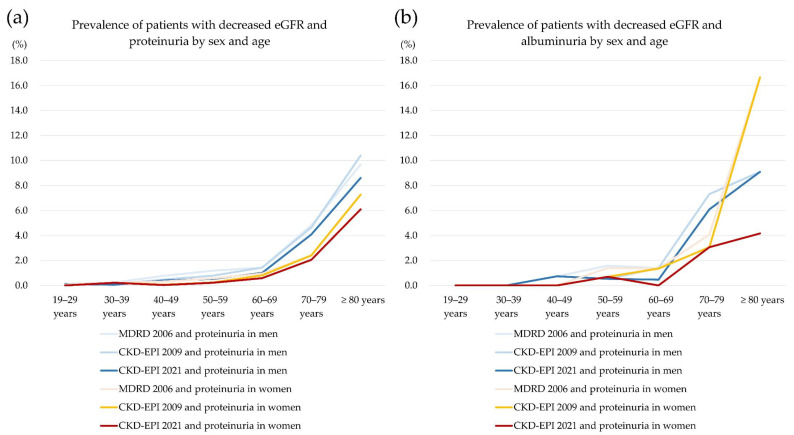
Prevalence of specimens with decreased eGFR (<60 mL/min/1.73 m^2^) and proteinuria (≥1+ in a dipstick urine protein test) or albuminuria (ACR > 30 mg/g creatinine) by sex and age.

The differences in the prevalence of decreased eGFR (≥G3a, <60 mL/min/1.73 m^2^) and proteinuria (≥1+) using a dipstick test or ≥ A2 albuminuria (urine ACR > 30 mg/g creatinine) by sex and age group are presented in Figure 4. Among age groups, those ≥80 years showed prominent differences in the prevalence. For sex groups, the prevalence of decreased eGFR (≥G3a, <60 mL/min/1.73 m^2^) with albuminuria in women aged ≥ 80 years was markedly changed from 16.7% when MDRD 2006 and CKD-EPI 2009 were applied to 4.2% when CKD-EPI 2021 was applied. The number of women aged ≥ 80 years was small, and the prevalence of decreased eGFR (≥G3a, <60 mL/min/1.73 m^2^) decreased to 14.5% with MDRD 2006, 14.4% with CKD-EPI 2009, and 11.6% with CKD-EPI 2021 after the data for women aged ≥ 80 years were combined with those aged 70–79 years.

## 4. Discussion

In this study, we investigated recent information on eGFR based on three equations (MDRD 2006, CKD-EPI 2009, and CKD-EPI 2021) and the specimen prevalence of possible CKD with decreased eGFR and proteinuria or albuminuria in an adult Korean population visiting local clinics and hospitals for health checkups.

Since the CKD-EPI 2021 without race information has been introduced, studies have been conducted for validation of its performance in different ethnic populations and various clinical settings, such as older acute medical patients, patients who underwent lung transplantation or kidney transplantation, and patients with solid tumors [8,11,12,13,14,15,16]. Previous studies reported the accuracy of CKD-EPI 2021 in different clinical settings [8,11,12,13,14,15,16]. However, there is a lack of studies regarding the effect of the equation changes in a Korean adult population.

In this study, eGFR decreased with increasing age, which confirmed previous findings [2]. This age-dependent decrease in eGFR increases the need for the use of age-specific thresholds for CKD [2]. However, recent studies have argued about the prognostic implications of an age-adapted definition of CKD [2,17]. Future studies are needed to clarify the need for and roles of age-specific eGFR thresholds in various ethnic populations [2].

In this study, a small proportion of specimens underwent simultaneous measurements of urine protein using dipstick tests, and the proportion of those with an ACR test was even smaller. The reason for the underutilization of the concurrent use of the assays may be due to the population characteristics, as the major population of this study was relatively healthy adult Koreans visiting local clinics and hospitals for health checkups. Another reason for the underutilization of ACR tests for albuminuria is that there are reimbursement conditions for the use of the test by the government (Health Insurance Review and Assessment Service, Korea). In Korea, ACR tests have been reimbursed when the dipstick protein test reveals no proteinuria in patients with diabetes, patients suspicious of diabetic nephropathy, or patients with hypertension and risks for cardiovascular complications (obesity, diabetes, hyperlipidemia, stroke, etc.) since 1 June 2018.

In this study, the overall agreement of eGFR grades of three categories ≤G2, G3a, and ≥G3b by CKD stage guidelines among three equations (MDRD 2006, CKD-EPI 2009, and CKD-EPI 2021) was 98.4% to 99.2%. However, the percentage of changes in specimen prevalence of decreased eGFR (≥G3a, <60 mL/min/1.73 m^2^) was higher than the percentage agreement of three eGFR grades among equations.

The calculated eGFR level based on the CKD-EPI 2021 equation was the highest, followed by the CKD-EPI 2009 and MDRD equations. The overall prevalence of decreased eGFR (<60 mL/min/1.73 m^2^) at baseline measurement was the highest based on the MDRD 2006 equation, followed by the CKD-EPI 2009 and CKD-EPI 2021 equations, which were comparable with previous findings performed in other ethnic populations (non-black persons) and KNHANES 2019 data [5,8]. According to the KNHANES 2019 data, the prevalence of decreased eGFR < 60 mL/min/1.73 m^2^ was 17.5% for MDRD 2006, 3.9% for CKD-EPI 2009, and 2.8% for CKD-EPI 2021 equations, respectively. The different decreases in prevalence of decreased eGFR and possible CKD may be due to cohort characteristics. The major population of this study was adults aged 40–49 years who visited local clinics and hospitals for health checkups, while the population cohort for KNHANES 2019 was a statistically selected representative population of Korea considering age, sex, and geographical and social status.

The lack of clinical information associated with CKD and decreased eGFR, such as comorbidities including hypertension, diabetes, cardiovascular disease, family history of CKD, acute renal damage, infection, obstruction, pregnancy, autoimmune disease, single kidney, ultrasound examinations of the urinary system to exclude renal abnormalities, and hematologic analysis of blood specimens to exclude dehydration, is a limitation of the present study. The aim of this study was to evaluate the prevalence of change by equations, and the accuracy of equations could not be evaluated in this study. Although the CKD should be examined with a decreased glomerular filtration rate (GFR, <60 mL/min/1.73 m^2^) for >3 months, in this study, almost 90% of the specimens were only examined once. Because the study population was Korean adults visiting local clinics and hospitals for health checkups and cystatin C measurement is not included in the national health checkup program in Korea, other equations for eGFR calculations using cystatin C were outside the scope of this study. However, the specimen prevalence of decreased eGFR, proteinuria, and albuminuria suggesting possible CKD in a large number of adult Koreans visiting local clinics and hospitals for health checkups with a recent long study period are the strengths of the present study and it might help to estimate the disease burden of CKD in general in adult Koreans.

## 5. Conclusions

In conclusion, this study investigated the effects of three equations to assess eGFR (MDRD 2006, CKD-EPI 2009, and CKD-EPI 2021) on the prevalence of possible CKD and the utilization of tests for proteinuria using dipstick reagent urinalysis protein tests and albuminuria using ACR tests in adult Koreans visiting local clinics and hospitals for health checkups. The eGFR decreased with increasing age, confirming previous findings and demonstrating that additional assessment of proteinuria or albuminuria is required, especially in older adults. The overall prevalence of decreased eGFR was significantly different among equations. The prevalence of CKD using eGFR based on CKD-EPI 2021 may result in a seeming decreased seroprevalence. Future studies regarding the impact on clinical outcomes of different equations are needed.

## Figures and Tables

**Table 1:** Baseline characteristics of 442,566 study subjects visiting local clinics and hospitals for health checkups.

Characteristics	Data
Age, year (Mean, SD)	50.1 (15.0)
Age group (*n*, %)	
19–29 years	46,882 (10.6)
30–39 years	73,217 (16.5)
40–49 years	105,633 (23.9)
50–59 years	92,879 (21.0)
60–69 years	76,935 (17.4)
70–79 years	37,313 (8.4)
≥ 80 years	9707 (2.2%)
SEX (*n*, %)	
Men	205,236 (46.4)
Women	237,330 (53.6)
Follow-up number (Mean, SD)	1.1 (0.40)
Serum creatinine (mg/dL, Mean, SD)	0.8 (0.33)
eGFR using MDRD 2006 (mL/min/1.73 m^2^, Mean, SD)	92.2 (19.8)
eGFR using CKD-EPI 2009 (mL/min/1.73 m^2^, Mean, SD)	97.1 (17.5)
eGFR using CKD-EPI 2021 (mL/min/1.73 m^2^, Mean, SD)	100.8 (17.1)
Decreased eGFR (<60 mL/min/1.73 m^2^)	
Based on MDRD 2006 (*n*, %)	15,208 (3.4%)
Based on CKD-EPI 2009 (*n*, %)	12,482 (2.8%)
Based on CKD-EPI 2021 (*n*, %)	9076 (2.1%)
Urine dipstick reagent strip protein test performed (*n*, %)	26,608 (6.0%)
Urine albumin-creatinine ratio test performed (*n*,%)	1151 (0.3%)
Both dipstick and albumin-creatinine ratio test performed (*n*, %)	319 (0.1%)

Abbreviations: SD, standard deviation; eGFR, estimated glomerular filtration rate.

**Table 2:** Overall agreement of qualitative estimated glomerular filtration rates (eGFR) grades among three equations for estimated glomerular filtration rates.

Agreement of Three Categories of eGFR; ≤G2, G3a, and ≥G3b)	*n* (%)
MDRD 2006 eGFR = CKD-EPI 2009 eGFR	439,101 (99.2%)
MDRD 2006 eGFR > CKD-EPI 2009 eGFR	325 (0.1%)
MDRD 2006 eGFR < CKD-EPI 2009 eGFR	3140 (0.7%)
MDRD 2006 eGFR = CKD-EPI 2021 eGFR	435,600 (98.4%)
MDRD 2006 eGFR > CKD-EPI 2021 eGFR	0 (0.0%)
MDRD 2006 eGFR < CKD-EPI 2021 eGFR	6966 (1.6%)
CKD-EPI 2009 eGFR = CKD-EPI 2021 eGFR	438,415 (99.1%)
CKD-EPI 2009 eGFR > CKD-EPI 2021 eGFR	0 (0.0%)
CKD-EPI 2009 eGFR < CKD-EPI 2021 eGFR	4151 (0.9%)

Abbreviations: SD, standard deviation; eGFR, estimated glomerular filtration rate. Qualitative categories for decreased eGFR are as follows: ≤G2 (normal to mild decrease, ≥60 mL/min/1.73 m^2^), G3a (mild to moderate decrease, 45–59 mL/min/1.73 m^2^), and ≥G3b (≥moderate decrease, <45 mL/min/1.73 m^2^).

**Table 3:** Overall agreement between dipstick proteinuria (equal or greater than 1+), urine albumin-creatinine ratio, and grade of estimated glomerular filtration rates (eGFR).

eGFR Grade		Urine Dipstick Protein Tests (*n* = 29,838)		Urine Albumin-Creatinine Ratio Tests (*n* = 1550)		
		Negative or Trace	≥1+	A1 (<30 mg/g Cr)	A2 (30–300 mg/g Cr)	A3 (>300 mg/g Cr)
Total		24,372 (91.6%)	2236 (8.4%)	917 (79.7%)	277 (19.7%)	7 (0.6%)
MDRD 2006 eGFR	≤G2 (≥60 mL/min/1.73 m^2^)	23,636 (96.8%)	1932 (86.4%)	863 (94.1%)	202 (89.0%)	6 (85.7%)
	G3a (45–59 mL/min/1.73 m^2^)	640 (2.6%)	188 (8.4%)	43 (4.7%)	20 (8.8%)	0 (0.0%)
	≥G3b (<45 mL/min/1.73 m^2^)	129 (0.5%)	116 (5.2%)	11 (1.2%)	5 (2.2%)	1 (14.3%)
CKD-EPI 2009 eGFR	≤G2 (≥60 mL/min/1.73 m^2^)	23,755 (97.5%)	1980 (88.6%)	874 (95.3%)	206 (90.7%)	6 (85.7%)
	G3a (45–59 mL/min/1.73 m^2^)	497 (2.0%)	140 (6.3%)	33 (3.6%)	16 (7.0%)	0 (0.0%)
	≥G3b (<45 mL/min/1.73 m^2^)	120 (0.5%)	116 (5.2%)	10 (1.1%)	5 (2.2%)	1 (14.3%)
CKD-EPI 2021 eGFR	≤G2 (≥60 mL/min/1.73 m^2^)	23,939 (98.2%)	2030 (90.8%)	888 (96.8%)	214 (94.3%)	6 (85.7%)
	G3a (45–59 mL/min/1.73 m^2^)	355 (1.5%)	111 (5.0%)	23 (2.5%)	10 (4.4%)	0 (0.0%)
	≥G3b (<45 mL/min/1.73 m^2^)	78 (0.3%)	95 (4.2%)	6 (0.7%)	3 (1.3%)	1 (14.3%)

Abbreviations: Cr, creatinine.

## Data Availability

The datasets generated and analyzed during the current study are available from the corresponding authors on reasonable request.

## Supplementary Materials

The following supporting information can be downloaded at: https://www.mdpi.com/article/10.3390/jcm11185339/s1, Table S1: Equations for estimated glomerular filtration rate (eGFR); Figure S1: Comparison of eGFR values between CKD-EPI 2009 and CKD-EPI 2021 equations. (a) Difference and (b) % difference in eGFR values between CKD-EPI 2009 and CKD-EPI 2021 equations. Horizontal lines represent mean difference and mean % difference, and dashed lines represents 95% confidence interval of mean difference and mean % difference.
