# Supplementary material for: Alterations in Glomerular Filtration Rates Using Different Algorithms in the Korean Population Visiting Local Clinics and Hospitals
Source: J Clin Med. 2022 Sep 11;11(18):5339. doi: 10.3390/jcm11185339 (PMC9501202; doi:10.3390/jcm11185339)
Supplement: Supplementary file 1 [file jcm-11-05339-s001.zip › jcm-1829607-supplementary.pdf]

Change in the Prevalence of Chronic Kidney Disease by Different Equations for Estimated Glomerular Filtration Rates in the Korean Population Visiting Local Clinics and Hospitals

Supplementary Table S1. Equations for estimated glomerular filtration rate (eGFR)

Abbreviation	Equation	Reference
MDRD 2006	eGFR (mL/min/1.73 m²) = 175 × $(Scr)^{-1.154}$ × $(Age)^{-0.203}$ × (0.742 if female) × (1.212 if African American)	[1]
CKD-EPI 2009	eGFR (mL/min/1.73 m²) = 141 × min$(Scr/k, 1)^{\alpha}$ × max$(Scr/k, 1)^{-1.209}$ × 0.993^{Age} × (1.018 if female) × (1.159 if African American), where k is 0.7 for females and 0.9 for males, α is -0.329 for females and -0.411 for males, min indicates the minimum of Scr/k or 1, and max indicates the maximum of Scr/k or 1.	[2]
CKD-EPI 2021	eGFR (mL/min/1.73 m²) = 142 × min$(Scr/k, 1)^{\alpha}$ × max$(Scr/k, 1)^{-1.200}$ × 0.9938^{Age} × (1.012 if female), where k is 0.7 for females and 0.9 for males, α is -0.241 for females and -0.302 for males, min indicates the minimum of Scr/k or 1, and max indicates the maximum of Scr/k or 1.	[3]

Abbreviations: MDRD, Modification of Diet in Renal Disease study equation; CKD-EPI, Chronic Kidney Disease Epidemiology Collaboration study equation; Scr, serum creatinine in mg/dL that assayed using methods traceable to isotope dilution mass spectrometry assigned and certified by the National Institute of Standards and Technology reference materials.

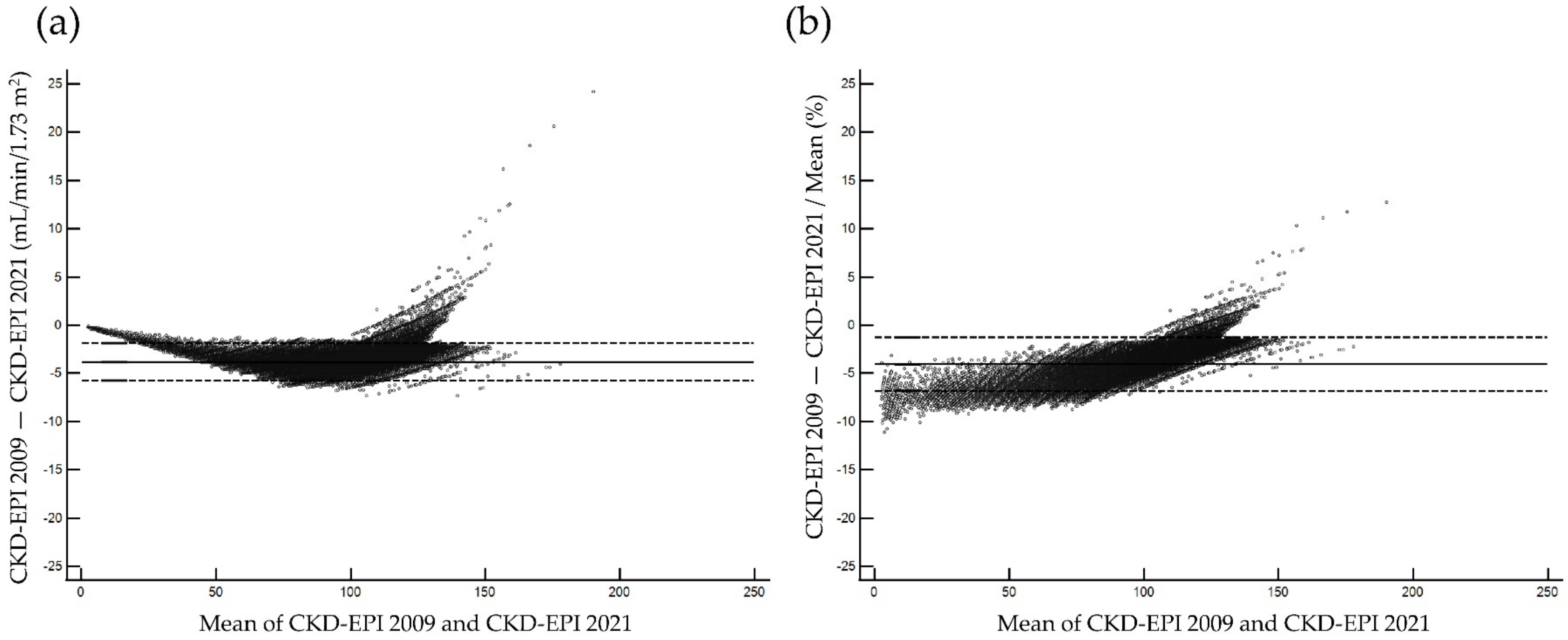

Figure S1. Comparison of eGFR values between CKD-EPI 2009 and CKD-EPI 2021 equations. (**a**) Difference and (**b**) % difference in eGFR values between CKD-EPI 2009 and CKD-EPI 2021 equations. Hori-zontal lines represent mean difference and mean % difference, and dashed lines represents 95% confidence interval of mean difference and mean % difference.

References

1. Levey, A.S.; Coresh, J.; Greene, T.; Stevens, L.A.; Zhang, Y.L.; Hendriksen, S.; Kusek, J.W.; Van Lente, F. Using standardized serum creatinine values in the modification of diet in renal disease study equation for estimating glomerular filtration rate. *Ann Intern Med* **2006**, *145*, 247-254.
2. Levey, A.S.; Stevens, L.A.; Schmid, C.H.; Zhang, Y.L.; Castro, A.F., 3rd; Feldman, H.I.; Kusek, J.W.; Eggers, P.; Van Lente, F.; Greene, T., et al. A new equation to estimate glomerular filtration rate. *Ann Intern Med* **2009**, *150*, 604-612.
3. Inker, L.A.; Eneanya, N.D.; Coresh, J.; Tighiouart, H.; Wang, D.; Sang, Y.; Crews, D.C.; Doria, A.; Estrella, M.M.; Froissart, M., et al. New Creatinine- and Cystatin C-Based Equations to Estimate GFR without Race. *N Engl J Med* **2021**, *385*, 1737-1749.
